# Mental health problems and suicidal behavior from adolescence to young adulthood in college: linking two population-based studies

**DOI:** 10.1007/s00787-023-02167-y

**Published:** 2023-02-27

**Authors:** Børge Sivertsen, Rory C. O’Connor, Sondre Aasen Nilsen, Ove Heradstveit, Kristin Gärtner Askeland, Tormod Bøe, Mari Hysing

**Affiliations:** 1 Department of Health Promotion, Norwegian Institute of Public Health, Bergen, Norway; 2 Department of Research and Innovation, Helse-Fonna HF, Haugesund, Norway; 3 Suicidal Behaviour Research Laboratory, Institute of Health and Wellbeing, University of Glasgow, Glasgow, UK; 4 Regional Centre for Child and Youth Mental Health and Child Welfare, NORCE Norwegian Research Centre, Bergen, Norway; 5 Department of Psychosocial Science, Faculty of Psychology, University of Bergen, Bergen, Norway

**Keywords:** Epidemiology, Prospective, Mental health, Deliberate self-harm, Suicide

## Abstract

It is well established that mental health problems are highly recurrent and persistent from childhood to adolescence, but less is known to what extent mental health problems also persist from adolescence into young adulthood. The aim of the current study was therefore to examine the chronicity and risk of mental health problems and suicidality from adolescence to young adulthood. Data stem from two Norwegian population-based studies conducted 6 years apart; the youth@hordaland study from 2012 (age 16–19) and the SHoT2018 study (age 22–25). These two data sources were linked to produce a longitudinal sample of 1257 individuals. A wide range of self-reported mental health and suicidality instruments (used both continuously and categorically) were analyzed using log-link binomial regression analysis, adjusting for age, sex, parental education, and financial problems. We found that high levels of mental health problems in late adolescence were a significant risk factor for reporting poor mental health 6 years later. Internalizing and externalizing problems in adolescence were associated with a 2.8-fold and 1.9-fold increased risk, respectively, of reporting a mental disorder 6 years later. Similarly, self-harm in adolescence was associated with a 2.1-fold increased risk of suicidal thoughts 6 years later. The magnitudes of the adjusted risk ratios were generally similar across the various mental health and suicidality measures used at the two assessment points. Adjustment for confounders did not, or only slightly, attenuate the risk ratios, and all associations remained statistically significant in the adjusted analyses. This longitudinal study provides new evidence of the chronicity of mental health problems and suicidality from adolescence to adulthood in Norway. The results emphasize the importance of early identification and timely interventions to reduce the prevalence and impact of mental health problems and suicidality.

**Supplementary Information:**

The online version contains supplementary material available at 10.1007/s00787-023-02167-y.

## Introduction

The transition from adolescence to adulthood is a time of substantial change and may also be a vulnerable period for the development of mental health problems [1]. Evidence from the last decade shows a disturbing rise in mental health problems on college campuses [2]. Increasingly, more students report that they are struggling from a wide array of mental health concerns, ranging from symptoms of psychological distress [2], to the more serious outcomes of non-suicidal self-harm (NSSH) and suicidal thoughts [3]. There is also evidence of a similar increase in mental disorders at a diagnostic level, with results from the WHO World Mental Health Surveys now showing that one in five college students fulfills the criteria for a 12-month DSM-IV disorder [4]. If left undetected and untreated, these mental health problems may cause considerable burden on an individual level, in addition to substantial societal consequences, including economic costs related to lost productivity from reduced workforce participation, as well as to government support payments [5]. 

The high and increasing prevalence of college mental health problems, combined with the wide range of consequences, makes it vital to identify the extent to which the mental health problems have their onset before college, as we know that increased mental health problems are present already in adolescence. Indeed, it is well established that, for some, mental disorders are highly recurrent and persistent from childhood to adolescence [6, 7]. However, there has been less focus on the transition from adolescence to young adulthood, but some longitudinal studies indicate that mental health problems to a large extent may persist from adolescence into adulthood [8–10]. For example, the longitudinal BELLA study from Germany reported that among children and adolescents aged 7–17 with mental health problems, and about one-third (31.5%) still were identified as having mental health problems 6 years later (ages 13–26) [11]. A similar level of persistence was found in the E-Risk Longitudinal Twin Study in the UK, where 22% of children with ADHD (enrolled at age 5–12) still fulfilled the diagnostic criteria for AHDH at age 18 [12]. Similarly, a US longitudinal study of 1420 participants found that children with a childhood mental disorder significantly predicted both suicidality and multiple psychiatric in young adulthood [13] Outcomes of childhood conduct problem trajectories in early. Also, findings from the population-based ALSPAC study have found childhood conduct problem to be pervasive and substantially affecting adjustment in young adulthood [14]. In contrast, a population-based Swiss study of 591 individuals recruited at age 19–20 found that the only a small proportion experienced persistent mental disorder later in of adulthood [15]. A similar pattern has also been observed for suicidal thoughts and behaviors, where a longitudinal study from New Zealand found suicidality in adolescence to predict both suicidal behaviors and major depression and anxiety disorder in young adulthood (18–25 years) [16]. However, given the dearth of research, there is still an urgent need for more longitudinal investigations to examine to what extent different mental health problems in adolescence represent risk factors for such difficulties as young people move into higher education.

Furthermore, we also know little about whether or not the increased risk of mental health problems represents a generalized risk factor for later mental ill health (*homotypic continuities)*, or if there are specific patterns related to different types of mental health problems (*heterotypic continuity)* [17, 18]. Caspi and Mofffit [19] have argued that mental disorders may often change into another condition, a liability which has been coined the p-factor of psychopathology. The observation that children and adolescents who report specific mental health problems also may suffer from other problems, was also the conclusion by Allegrini and colleagues, employing a large twin study of 7,026 twin pairs [20]. However, the authors of these studies emphasized that there is still a strong need for longitudinal studies to further examine this, as most studies in this field are cross-sectional. Furthermore, there is still a research gap focusing specifically on the transition from adolescence to young adulthood.

In light of these considerations, the overall aim of the current longitudinal study was to examine to what extent different measures of mental health problems in adolescents aged 16–19 years were associated with increased mental health problems 6 years later, at age 22 to 25 years. A secondary aim was to explore to what extent the observed associations represented homotypic continuities (continuation within one domain of problem/disorder), or heterotypic continuity (where there is continuation from one domain of disorders into another).

## Methods

### Participants

Data stem from two linked population-based studies conducted 6 years apart, the youth@hordaland study (Y@H; T1) from 2012 and the SHoT2018 study. The Y@H was conducted in the winter/spring of 2012 and included all adolescents in Hordaland County. Hordaland is fairly representative of Norway, comprising both urban areas (incl. Bergen, the second largest city in Norway) and large rural areas. In all, 10,257 of the 19,439 invited adolescents participated, yielding a response rate of 53%.The Y@H study has been detailed in previous publications (e.g., [21]).

The SHoT2018 study (Students’ Health and Wellbeing Study; T2) is a national student survey for higher education in Norway. The SHoT2018 was collected electronically through a web-based platform. Details of the study have been published elsewhere [22]. In short, the SHoT2018 was conducted between February 6 and April 5, 2018, and invited all fulltime Norwegian students pursuing higher education (both in Norway and abroad). In all, 162,512 students fulfilled the inclusion criteria (being a fulltime student aged 18 years or older), of whom 50,054 students completed the online questionnaires, yielding a response rate of 31%. Participants received no payment for participation.

When consenting to participate in the SHoT2018 study, participants were also asked if their SHoT data could be linked to the regional Y@H study, for those who took part in that specific study 6 years earlier. In all, 1,259 adolescents were eligible and consented to this linkage, which comprises the current study sample.

Individuals who took part in both the Y@H study and SHoT2018 studies (responders) were more likely to have parents with higher education. This was expected given that parent education levels are strongly associated with offspring education levels in Norway. Therefore, we would expect students in higher education to have parents who also have higher education, relative to the general population. There were no differences in terms of sex and perceived economic wellbeing, compared with individuals who did not complete both assessments (non-responders) (see Supplementary Table for more details).

### Instruments

#### Sociodemographic information

Sex and age were identified through the personal identity numbers in the Norwegian National Population Register. Given the age span in the Y@H (16–19 years) and time between the two data collections (6 years), only participants aged 22–25 from the SHoT2018 were included in the present study (representing the age cohorts for the Y@H). Socioeconomic status (SES) was assessed by parental education and perceived economic wellbeing. Maternal and paternal education (highest level) was reported separately with three response options; “primary school”, “secondary school”, and “college or university”. Perceived economic wellbeing (i.e., how well off the adolescent perceived their family to be) was assessed by asking the adolescents how their financial circumstances were compared to most others. Response alternatives were 1) “better than others”, 2) “equal to others”, and 3) “poorer than others”.

### Instruments

Both studies were approved by the Regional Committee for Medical and Health Research Ethics in Western Norway. For both studies, an electronic informed consent was obtained after the participants had received a detailed introduction to the study. Participants received no payment for participation. For the Y@H, the adolescents’ parents were informed about the study, while the adolescents themselves consented to participate in the study as Norwegian regulations state that individuals aged 16 years and older are required to consent themselves. The list of instruments included both continuous and categorical/dichotomous measures at both time points.

### Predictors at T1

#### Internalizing and externalizing problems

Internalizing and externalizing problems were measured by the Strengths and Difficulties Questionnaire (SDQ). The SDQ is a screening instrument for mental health problems initially developed for children and adolescents aged 4–17 years [23, 24]. It consists of five subscales measuring emotional symptoms, conduct problems, hyperactivity–inattention, peer-relationship problems, and prosocial behaviors. Each subscale consists of five items, measured on a 3-point Likert scale (‘not true’, ‘somewhat true’, or ‘certainly true’). The internalizing problems scale is created by combining the peer problems and emotional problems subscales, whereas the general externalizing problems scale is created by combining the conduct problems and hyperactivity–inattention subscales [25]. Previous investigations have found the SDQ to be reliable and valid for use in samples of adolescents up to 19 years of age [26], and a previous study found that the SDQ displayed adequate psychometric properties also in the current sample of older Norwegian adolescents from the Y@H [27]. The SDQ internalizing and externalizing subscales were both used continuously and dichotomized at the 90^th^ percentile to indicate high scorers. The cut-off point for clinical range is usually recommended to be roughly above the 90^th^ percentile of SDQ scores [28].

#### Symptoms of depression

Symptoms of depression were assessed using the short version of the Mood and Feelings Questionnaire (SMFQ) [29]. The SMFQ comprises 13 items assessing depressive symptoms rated on a three-point Likert scale. The wording of the response categories in the Norwegian translation equate to the original categories of “not true”, “sometimes true”, and “true”. High internal consistency between the items and strong unidimensionality have been shown in population-based studies [30], and these have been confirmed in a Norwegian study based on the sample included in the present study [31]. The SMFQ was both used continuously and dichotomized at the 90th percentile to indicate high scorers. While no clinical cut-offs exist for the SMFQ, a total score above the 90th percentile has previously been used as an operationalisation of depression [30].

#### Symptoms of anxiety

Symptoms of anxiety were identified using the short five-item version of the Screen for Child Anxiety Related Emotional Disorders (SCARED) [32]. This short version consists of five items found to discriminate between anxious and non-anxious children and has similar psychometric properties to the full 41-item SCARED [32]. The items are rated on a 3-point Likert scale, with the options ‘0—not true’, ‘1—sometimes true’, and ‘2—often true’ (range 0 to 10). The SCARED was both used continuously and dichotomized at the 90th percentile to indicate high scorers. A cut-off of 3 is the recommended cut-off for discriminating anxiety from nonanxiety [32], which in the current study corresponded to the 90^th^ percentile.

#### Attention-deficit/hyperactivity disorder (ADHD) symptoms

ADHD symptoms were measured using the Adult ADHD Self-Report Scale (ASRS) [33]. ASRS consists of 18 items: nine items measure inattention symptoms, and nine items measure symptoms of hyperactivity–impulsivity. Although initially developed for adults, the ASRS has been validated and found to have high internal consistency and construct validity among adolescents [34]. Symptoms are rated on a 5-point Likert scale ranging from ‘never’ to ‘very often’. The ASRS was both used continuously and dichotomized at the 90th percentile to indicate high scorers (which is also the recommended clinical cutoff [34]).

#### Obsessive–compulsive disorder (OCD) symptoms

Five items measured key aspects of OCD, as outlined by Thomsen [35]: ‘I wash myself more than normal. I am afraid of infection’, ‘I often have to check or control things’, ‘I am concerned with order and symmetry’, ‘I must often have repeated assurances and answers to questions’, and ‘I have distressing or disturbing thoughts’. The items were rated on a 3-point Likert scale (‘not true’, ‘somewhat true’, and ‘certainly true’). The OCD measure was both used continuously and dichotomized at the 90th percentile to indicate high scorers (no clinical cutoff has been published for the instrument).

#### Self-harm

Self-harm was assessed using the following question taken from the Child and Adolescent Self-harm in Europe (CASE) Study [36]: “Have you ever deliberately taken an overdose (e.g., of pills or other medication) or tried to harm yourself in some other way (such as cut yourself)?” Classification of self-harm was done according to the CASE guidelines by two coders and in line with the CASE definition of self-harm: *“act with a non-fatal outcome in which an individual deliberately did one or more of the following: initiated behaviour (e.g., self-cutting, jumping from a height), which they intended to cause self-harm; ingested a substance in excess of the prescribed or generally recognized therapeutic dose; ingested a recreational or illicit drug that was an act the person regarded as self-harm; ingested a non-ingestible substance or object”.* More details on the self-harm measure in the Y@H have been published elsewhere [37].

### Outcome variables at T2

#### Psychological distress

Psychological distress was assessed using The Hopkins Symptoms Checklist (HSCL-25) [38], derived from the 90-item Symptom Checklist (SCL-90), a screening tool designed to detect symptoms of anxiety and depression. It is composed of a 10-item subscale for anxiety, and a 15-item subscale for depression, with each item scored on a Likert scale from 1 (“*not at all*”) to 4 (“*extremely*”). The reference period is the previous 2 weeks. Several factor structures and cut-offs for clinical levels have been proposed for the HSCL-25 [39, 40]. An investigation of the factor structure based on the SHoT2014 dataset from Norway showed that a unidimensional model had the best psychometric properties in the student population and not the original subscales of anxiety and depression [41]. We have chosen to follow this recommendation in the present study. An average score on the HSCL-25 of > 2.00 was used as the cut-off value for identifying a high level of mental health problems. Details on the development of mental health problems in the SHoT waves were recently published by Knapstad et al. [2]. In the current study, the HSCL-25 was used both continuously and dichotomously.

#### Anxiety or depressive disorder

Self-reported mental disorders were assessed by a pre-defined list adapted to fit this age-cohort. The list was based on a similar operationalisation used in previous large population-based studies (the HUNT study [42]) and included several subcategories for most conditions/disorders (not listed here). The list contained no definition of the included disorders/conditions. Due to statistical power limitations, the current study included only anxiety or depressive disorder (combined).

#### Non-suicidal self-harm and suicidal thoughts

History of non-suicidal self-harm (NSSH) and suicidal thoughts were assessed with two items drawn from the Adult Psychiatric Morbidity Survey (APMS) [43]; *“Have you ever deliberately harmed yourself in any way but not with the intention of killing yourself? (i.e., self-harm)”*, and *“Have you ever seriously thought of taking your life, but not actually attempted to do so?”* If respondents answered yes to any item, the timing of the most recent episode was assessed, using the following response options: “last week”, “past year”, “more than a year ago, but after I started studying at the university”, and “before I started studying at university”. More detailed information about self-harm and suicidal behavior in SHoT2018 has been published elsewhere [44].

### Statistics

**Fig. 1:**
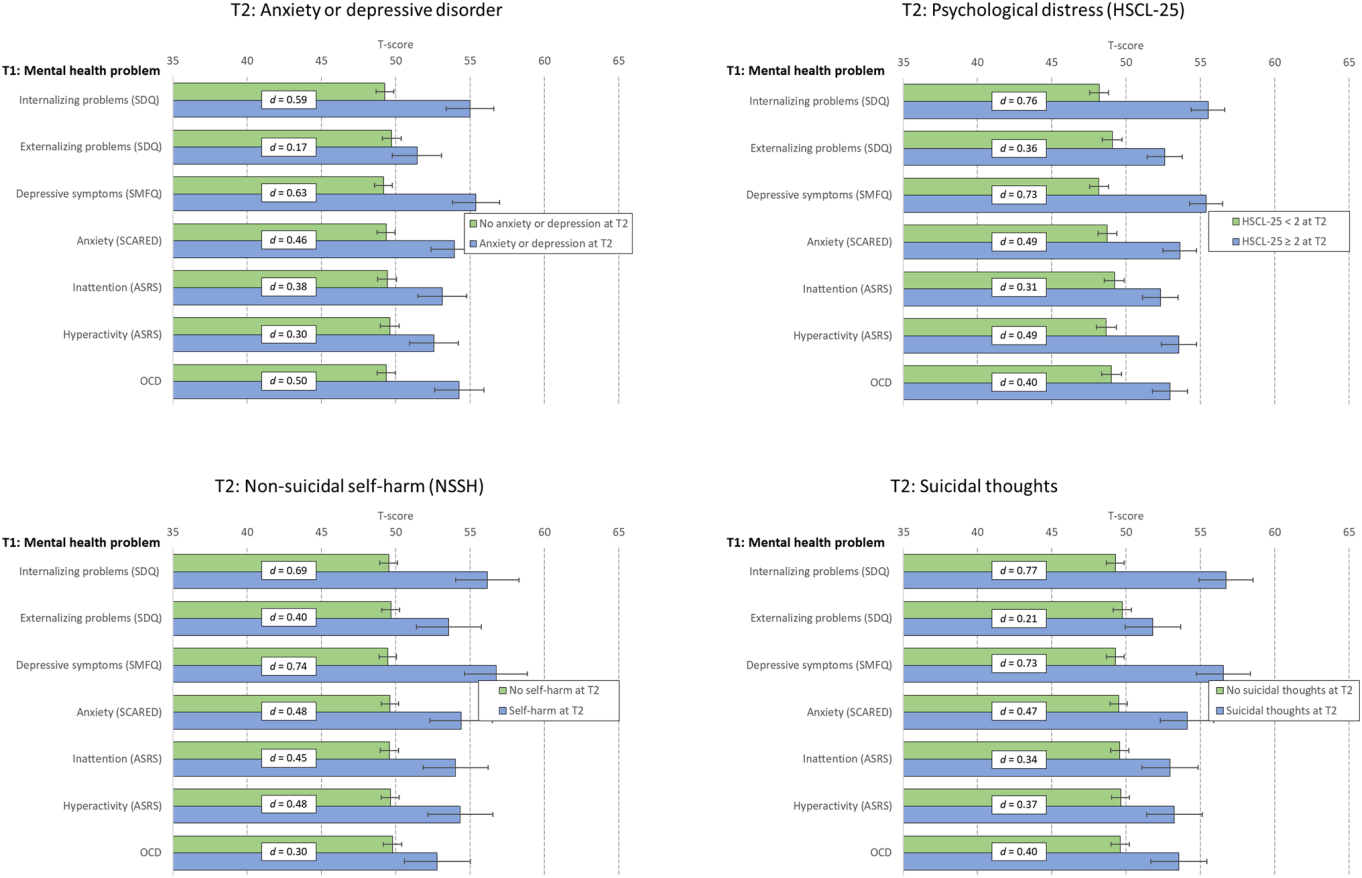
Differences in mental health problems (continuous measures) at T1 by mental health, self-harm, and suicidal thoughts at T2 (dichotomous measures), represented in adjusted T-scores (in bars) and Cohen’s d effect size (in white text box). Error bars represent 95% confidence intervals

IBM SPSS version 27 (SPSS Inc., Chicago, IL, United States) for Windows was used for all analyses. For all continuous measures (SDQ, SMFQ, SCARED, ASRS, and OCD—displayed in Fig. 1), sum scores were converted to standardized t-scores to ease the comparison across the instruments, and between-group effect sizes (pooled SD) were calculated using the Cohen d formula. The effect sizes can be interpreted according to Cohen’s guidelines, with d’s of about 0.20 representing small effect sizes, d’s of about 0.50 medium effect sizes, and d’s greater than 0.80 representing large effect sizes. Pearson chi-squared tests were used to compare the prevalence of mental health problems at T2 by low/high level of mental health problems at T1, and log-link binomial regression analyses were used to calculate risk ratios (RR), adjusting for age, sex, parental education, and perceived economic wellbeing (at T1), as detailed in Table 1. There was generally little missing data, and hence, missing values were handled using listwise deletion. No a priori power calculations were conducted to ensure that the sample size had sufficient statistical power to detect differences in outcomes, as both the Y@H and the SHoT study had several objectives and were not designed to be a study of these associations specifically.

**Table 1:** Prevalence and adjusted risk of mental health problems and suicidality at T2 (SHOT 2018) by mental health problems in T1

T2: variable (SHOT2018), % (*n*)	T1: internalizing problems (SDQ)						T1: externalizing problems (SDQ)					
	Low SDQ		High SDQ^#^		Adj. RR^$^	(95% CI)	Low SDQ		High SDQ^#^		Adj. RR	(95% CI)
Anxiety or depressive disorder	11.1%	(118)	35.6%	(26)	2.84***	1.99–4.06	11.9%	(126)	26.1%	(18)	1.90**	1.22–2.95
Psychological distress (HSCL25 > 2)	22.4%	(237)	60.3%	(44)	2.38***	1.96–2.94	23.4%	(248)	47.8%	(33)	1.83***	1.39–2.40
Non-suicidal self-harm (NSSH) ^§^	6.0%	(64)	20.5%	(15)	3.18***	1.91–5.28	5.8%	(62)	24.6%	(17)	3.95**	2.44–6.37
Suicidal thoughts^§^	8.3%	(88)	28.8%	(21)	3.12***	2.04–4.76	8.8%	(94)	21.7%	(15)	2.25**	1.35–3.74

T2: variable (SHOT2018), % (*n*)	T1: depressive symptoms (SMFQ)						T1: anxiety symptoms (SCARED)					
	Low SMFQ		High SMFQ^#^		Adj. RR	(95% CI)	Low SCARED		High SCARED^#^		Adj. RR	(95% CI)
Anxiety or depressive disorder	11.1%	(111)	30.2%	(32)	2.29***	1.62–3.25	12.0%	(139)	24.3%	(25)	1.72**	1.16–2.55
Psychological distress (HSCL25 > 2)	21.5%	(216)	58.5%	(62)	2.22***	1.82–2.72	23.1%	(243)	50.7%	(38)	1.75***	1.36–2.26
Non-suicidal self-harm (NSSH)	5.5%	(55)	21.7%	(23)	3.20***	2.03–5.06	6.4%	(67)	16.0%	(12)	2.10*	1.19–3.72
Suicidal thoughts	7.9%	(79)	25.5%	(27)	2.73***	1.83–4.10	8.8%	(93)	21.3%	(16)	2.07***	1.25–3.41

T2: variable (SHOT2018), % (*n*)	T1: hyperactivity (ASRS)						T1: inattention (ASRS)					
	Low ASRS Hyp		High ASRS Hyp^#^		Adj. RR	(95% CI)	Low ASRS Inatt		High ASRS Inatt^#^		Adj. RR	(95% CI)
Anxiety or depressive disorder	11.6%	(118)	26.9%	(25)	2.03***	1.39–2.97	10.5%	(105)	30.8%	(37)	2.72***	1.96–3.77
Psychological distress (HSCL25 > 2)	23.1%	(235)	46.2%	(43)	1.87***	1.47–2.37	21.3%	(212)	55.0%	(66)	2.35***	1.94–2.84
Non-suicidal self-harm (NSSH)	6.0%	(61)	19.4%	(18)	2.90***	1.80–4.67	5.7%	(57)	17.5%	(21)	2.65***	1.66–4.21
Suicidal thoughts	8.7%	(89)	19.4%	(18)	1.96**	1.21–3.14	8.1%	(81)	20.8%	(25)	2.31***	1.52–3.50

T2: variable (SHOT2018), % (*n*)	T1: OCD						T1: self-harm					
	Low OCD		High OCD^#^		Adj. RR	(95% CI)	No self-harm		Self-harm		Adj. RR	(95% CI)
Anxiety or depressive disorder	10.8%	(109)	29.2%	(35)	2.66**	1.93–3.67	11.6%	(122)	29.3%	(22)	1.92**	1.27–2.91
Psychological distress (HSCL25 > 2)	23.0%	(232)	40.8%	(49)	1.69***	1.34–2.14	23.2%	(268)	44.7%	(46)	1.64***	1.29–2.09
Non-suicidal self-harm (NSSH)	6.4%	(65)	11.7%	(14)	1.73*	1.01–2.97	6.0%	(69)	18.4%	(19)	2.67***	1.65–4.31
Suicidal thoughts	8.4%	(85)	20.0%	(24)	2.26***	1.49–3.43	9.2%	(106)	20.4%	(21)	2.10***	1.35–3.27

^$^RR = risk ratio (adjusted for age, sex, parental education, and financial problems)

^#^(90^th^ percentile)

^§^Estimates refer to participants having such thoughts and behaviors after started studying at college/university

*p < 0.05, ** *p* < 0.01, *** *p* < 0.001

## Results

### Sample characteristics

The longitudinal sample included in the present study consisted of 1,257 individuals. 69.7% of the participants were female, and the educational level of their parents was comparable to the national average for students in higher education [45]. 53.1% of the mothers and 48.3% of the fathers had an educational level higher than high school.

### Predictors of mental health problems, NSSH, and suicidal thoughts in adulthood

As detailed in Table 1, more symptoms of mental health problems in late adolescence (T1: age 16–19) were a significant risk factor for reporting poorer mental health 6 years later, at age 22–25 (T2). Although some variations in effect sizes (RRs) were observed, the magnitude of the adjusted RRs were generally similar across the mental health and suicidality measures used at the two assessment points. For example, adolescents with high levels of internalizing problems in adolescence had a 2.8-fold increased risk of later reporting anxiety or depressive disorder 6 years later, and similar RRs were found when using psychological distress, NSSH, and suicidal thoughts as the outcome measures. Adjusting for age, sex, parental education, and perceived economic wellbeing did not, or only slightly, reduce the strength of the associations. The same patterns were observed when examining the predictive effect of externalizing problems, depressive-, anxiety- and OCD symptoms, symptoms of ADHD, as well as self-harm in adolescence (see Table 1 for details). In terms of self-harm (which was similarly assessed at both assessment points), among the 8.2% who reported NSSH at T1, 18.4% also reported this at T2, 6 years later (adj. RR = 2.67, 95% CI 1.65–4.31).

### Mental health problems in adolescence by mental health status in adulthood

Figure 1 displays the scores on the mental health instruments at T1 (converted to T-scores) by mental health status at T2. College and university students with anxiety or depression at T2 had significantly higher levels of mental health problems across all instruments 6 years earlier, with the highest effect size observed for depressive symptoms (SMFQ: Cohen’s *d* = 0.63) and internalizing problems (SDQ: Cohen’s *d* = 0.57). Similar patterns and levels were observed for college and university students suffering from psychological distress, NSSH, and suicidal thoughts at T2 (see Fig. 1 for details).

As detailed in Table 2, the Spearman Rank Correlation analyses between the HSCL-25 (psychological distress) at T2 and all continuous mental health instruments at T1 showed that all correlations were highly significant at *p* < 0.001, ranging from rho = 0.209 (OCD) to rho = 0.413 (SDQ Internalizing problems) and rho = 0.441 (SMFQ depressive symptoms).

**Table 2:** Spearman rank correlation (Rho) between HSCL total score at T2 and instruments assessing mental health problems (T1)

	HSCL-25 Spearman’s rho
Internalizing problems (SDQ)	0.413***
Externalizing problems (SDQ)	0.229***
Depressive symptoms (SMFQ)	0.441***
Anxiety (SCARED)	0.317***
Hyperactivity (ASRS)	0.272***
Inattention (ASRS)	0.231***
OCD	0.209***

***p < 0.001

## Discussion

By linking two population-based studies conducted 6 years apart, the current results provide further evidence that mental health problems in late adolescence pose a significant risk for subsequent poor mental health in young adulthood. The observed associations were generally similar across the different mental health domains and adjusting for confounders had little impact.

The overall pattern of results suggests that mental health problems are a general risk factor for later mental ill health. The strong continuity in mental health problems over time supports findings from previous studies [46, 47]. There was also evidence of both *homotypic continuities*, such as adolescent who were high scorers on internalizing mental health problems (measured by the SDQ internalizing problems and specific measures of depressive and anxiety symptoms during adolescence), having increased risk of later internalizing symptoms in emerging adulthood. However, there was also some evidence of a *heterotypic continuity*, illustrated by a continuation from one domain of disorders into another, such as when adolescents reporting externalizing symptoms were at increased risk of internalizing mental health problems 6 years later. However, the assessment of heterotypic continuity was somewhat restricted by the measures in SHOT study, and the inclusion of instruments assessing ADHD and conduct problems during young adulthood, would have been beneficial in this regard.

For the continuous measures, the patterns were similar, although some differences in observed magnitudes across the various types of mental health problems were observed. For those who scored highly on internalizing problems/psychological distress during young adulthood, the effect sizes for internalizing problems were in the medium range, while the effect sizes for externalizing problems were in the small range, suggesting a somewhat stronger homotypic than heterotypic continuity. The findings that internalizing problems were more slightly stable compared to externalizing problems is a rather novel finding. While possible explanations for these findings may not be entirely clear, it could be related to the fact that the present study focused on college students specifically. Indeed, college students may have more internalizing problems, and not so much externalizing problems compared to the general population, which suggest that the generalizability in this study should be interpreted bearing this in mind.

The high stability of NSSH over time is in accordance with the few previous longitudinal studies from adolescence to young adulthood, as observed both in clinical [48] and population-based studies [49]. This is also consistent with the previously mentioned Swedish longitudinal study, where self-harm during adolescence predicted mental disorders in early adulthood [49]. The high correlation and chronicity among different domains of mental health disorders have been observed previously, and some researchers have suggested that mental health problems in emerging adulthood may best be characterized by one general factor as well as more specific externalizing and internalizing factors [50, 51].

Some study limitations should be noted. The large attrition rate and the nature of the sample may restrict generalisability to the general population [52]. The sample was also characterized by having higher educated parents relative to the general population, and we know that mental health problems are socioeconomically patterned [53]. Together, this suggests that the current findings may be a conservative estimate of mental health problems, and thus an underestimation of the prevalence of such problems in the general population of young adults. Finally, additional waves of data collection would have been useful to provide a more detailed picture of mental health trajectories from adolescence to young adulthood. It is also important to note that all constructs were measured differently across the time points, as a consequence of including age-appropriate instruments in each of the two studies.

Among the advantages of the study is the broad assessment of mental health, including several well-validated questionnaires at both time points. Also, we included and controlled for several relevant covariates, but none of these appeared to play a large role in the stability of mental health problems over time, as evidenced by the small attenuation in relative risk in the adjusted models. Finally, the repeated measures design is a strength, as well as a very large sample size and use of representative data.

The current study extends the limited knowledge on the stability of mental health problems from adolescence to young adulthood and confirms a pattern of stability over time. More studies are needed to further our understanding of the specific patterns of homotypic and heterotypic continuity, as well as indicated risk and resilience factors for the development of mental health problems from adolescence to young adulthood. Still, the results highlight the importance of early identification and timely interventions to reduce the prevalence and impact of mental health problems, both during the adolescence years and in young adulthood. Specifically, college and university students may be at particular risk for persistent internalizing problems and disorders, which indicate the need for early identification and adequate support for this group. More focus on preventive measures is obviously warranted by both universities and health authorities, to ensure health promoting and inclusive settings for our college and university students.

### Supplementary Information

Below is the link to the electronic supplementary material.

Supplementary file1 (DOCX 18 KB)
