# Supplementary material for: Mental health problems and suicidal behavior from adolescence to young adulthood in college: linking two population-based studies
Source: Eur Child Adolesc Psychiatry. 2023 Feb 27;33(2):421–9. doi: 10.1007/s00787-023-02167-y (PMC10869414; doi:10.1007/s00787-023-02167-y)
Supplement: Supplementary file 1 — Supplementary file1 (DOCX 18 KB) [file 787_2023_2167_MOESM1_ESM.docx]

| **Supplementary table 1.** Demographical variables in youth@hordaland and SHoT2018 studies (separate) and linked longitudinal sample (n=1259). | | | | |
| --- | --- | --- | --- | --- |
| Demographics at T3 | youth@hordaland only | SHoT2018 only | Both studies | p-value |
| N |  |  |  |  |
| Females | 50.7% (4,200) | 68.6% (28,463) | 69.7% (875) |  |
| Maternal education |  |  |  | <.001 |
| Primary school | 8.0% (655) |  | 5.9% (67) |  |
| High school | 31.7% (2,597) |  | 27.9% (316) |  |
| University/college | 34.5% (2,830) |  | 53.1% (602) |  |
| Paternal education |  |  |  | <.001 |
| Primary school | 8.0% (650) |  | 7.1% (81) |  |
| High school | 35.4% (2,897) |  | 28.8% (326) |  |
| University/college | 29.5% (2,410) |  | 48.3% (547) |  |
| Economic well-being |  |  |  | .426 |
| Equal to others | 67.4% (5454) |  | 68.0% (760) |  |
| Better to others | 25.5% (2062) |  | 25.9% (290) |  |
| Poorer to others | 7.1% (578) |  | 6.1% (68) |  |
